# Syphilis prevalence trends in adult women in 132 countries – estimations using the Spectrum Sexually Transmitted Infections model

**DOI:** 10.1038/s41598-018-29805-9

**Published:** 2018-07-31

**Authors:** Eline L. Korenromp, S. Guy Mahiané, Nico Nagelkerke, Melanie M. Taylor, Rebecca Williams, R. Matthew Chico, Carel Pretorius, Laith J. Abu-Raddad, Jane Rowley

**Affiliations:** 1 Avenir Health, Geneva, Switzerland; 2 Avenir Health, Glastonbury, CT USA; 3 Malawi-Liverpool Wellcome Trust, Blantyre, Malawi; 4 World Health Organization, Dept. of Reproductive Health and Research, Geneva, Switzerland; 5 Centers for Disease Control and Prevention, Division of STD Prevention, Atlanta, Georgia USA; 6 London School of Hygiene and Tropical Medicine, London, United Kingdom; 7 Weill Cornell Medical College - Qatar, Cornell University, Doha, Qatar; 8 Unaffiliated, London, United Kingdom

## Abstract

We estimated national-level trends in the prevalence of probable active syphilis in adult women using the Spectrum Sexually Transmitted Infections (STI) model to inform program planning, target-setting, and progress evaluation in STI control. The model fitted smoothed-splines polynomial regressions to data from antenatal clinic surveys and screening and representative household surveys, adjusted for diagnostic test performance and weighted by national coverage. Eligible countries had ≥1 data point from 2010 or later and ≥3 from 2000 or later from adult populations considered representative of the general female population (pregnant women or community-based studies). Between 2012 and 2016, the prevalence of probable active syphilis in women decreased in 54 (41%) of 132 eligible countries; this decrease was substantive (≥10% proportionally, ≥0.10% percentage-point absolute difference and non-overlapping 95% confidence intervals in 2012 and 2016) in 5 countries. Restricting eligible data to prevalence measurements of dual treponemal and non-treponemal testing limited estimates to 85 countries; of these, 45 countries (53%) showed a decrease. These standardized trend estimates highlight the need for increased investment in national syphilis surveillance and control efforts if the World Health Organization target of a 90% reduction in the incidence of syphilis between 2018 and 2030 is to be met.

## Introduction

Syphilis is an infection caused by the spirochete *Treponema pallidum*. It can be transmitted through sexual activity and from a mother to fetus vertically during pregnancy or newborn during childbirth. When untreated, syphilis causes substantial morbidity and mortality, not only in adults, but also in infants and young children as congenital syphilis.

Many countries have committed to reducing rates of adult syphilis, and to eliminating congenital syphilis. The World Health Organization (WHO) “Global Health Sector Strategy on Sexually Transmitted Infections (STIs) 2016–2021” has two syphilis related targets^1,2^: a 90% reduction in *Treponema pallidum* incidence globally between 2018 and 2030, and 50 or fewer cases of congenital syphilis per 100,000 live births in 80% of countries. Monitoring progress toward these targets is hampered by the quality and quantity of national data. Many national programs lack an estimate of their current burdens of adult and congenital syphilis, compromising not only progress evaluation but also target-setting, program planning, and costing^3^.

The WHO produces global and regional syphilis prevalence and incidence estimates for adult women roughly every four years. The most recent estimates were for 2012, when WHO estimated that the global prevalence of syphilis in adult men and women were 0.49% (0.4–0.6%) and 0.48 (0.3–0.7%) and there were 350,000 adverse pregnancy outcomes in infected pregnant women^4^. The Institute of Health Metrics and Evaluation (IHME) also estimated syphilis burdens^5^. However, to date, there has been no systematic exploration of country-level trends.

The Spectrum Sexually Transmitted Infections (STI) model (Spectrum-STI) was incorporated into the Spectrum suite of health policy models in 2016 as a tool that countries can use to estimate trends in the prevalence and incidence of syphilis, gonorrhea and chlamydia^6–8^. The model estimates national adult prevalence trends by fitting statistical models to prevalence data, adjusted for diagnostic test performance and weighted by national coverage.

In this paper, the Spectrum-STI model and the Spectrum syphilis database were used to examine trends over time in the prevalence of probable active syphilis in women 15 to 49 years of age between 2012 and 2016. The focus of the paper is on the methods and estimates of trends across countries and cross-country patterns in data availability, but not on the results for individual countries. The paper also explores how data eligibility criteria and statistical methods influence the estimates.

## Methods

### Spectrum-STI model

The basic structure of the Spectrum-STI model has been described^6^. Some refinements have been made to the model since the initial country applications:

1. Prevalence data are fitted to calendar year using segmented, second-order polynomial spline regressions^9–11^, with a maximum of two knots rather than a simple logistic regression (Supplementary Information 1(SI1)). This allows for up to three historic phases or trends in a country’s prevalence of syphilis.
2. Prevalence is fitted through the corresponding incidence rate (SI1). The constraint that incidence must be ≥0 in any year ensures that there are no unrealistic sharp falls in prevalence from one year to the next, which would be inconsistent with epidemiologically valid incidence rates and average duration of infection.
3. A random effects component has been included to account for heterogeneity across prevalence measurements within a country owing to the variability in prevalence measures due to variations in the sampled study population, other than sampling variation. Prevalence estimates were resampled following beta distributions, to which noise was added in the logit scale and simulated as a function of the population sampled to account for uncertainty and possible biases associated with pooling prevalence data across study populations (surveys in Antenatal Care clinics (ANC), routine ANC screening, adult women not in antenatal care, adult men, and adult men and women without sex disaggregation). This guards against over-interpreting fluctuations between successive data points reflecting unaccounted sources of variation, including reporting errors, in the data.
4. Prevalence time trends were extrapolated to two years after the latest national data point, and to two years before the first available national data point. For earlier and later years, prevalence was assumed to be constant at the earliest and latest estimates.

Infection episode durations, used to inform the relationship between incidence and prevalence, were taken as assumed in the WHO’s 2012 global syphilis estimations^4^: Weighted between treated and untreated episodes, the weighted duration was set at 1.28 years in countries with good STI treatment access (high income countries), 2.42 years in countries with moderate access (South and Central Americas and the Caribbean, Oceania, East and Central Asia, Central Europe, Eastern Mediterranean) and 4.13 years in countries with poor access (sub-Saharan Africa, and South and South-East Asia). Each of these values was assumed to have a standard error of ±50%.

As in the original 2016 model, estimated prevalence was constrained to a maximum value of 20% for all years and all countries to avoid extrapolations to overly high (and unrealistic) prevalence values. The 20% maximum value was based on available data from low- and middle-income countries since 1990^4,12^.

Statistical analyses were carried out in R version 3.4.0^13^. Bootstrapping (400 replications) was used to generate uncertainty bounds^6^. In each bootstrap iteration, three sets of variables were resampled: prevalence data, diagnostic test adjustment values, and duration of infection. The fitting procedure was applied to the simulated/bootstrapped data, and confidence intervals (CIs) were derived using the percentile method.

### Spectrum syphilis database

The Spectrum-STI global syphilis database is a comprehensive compilation of syphilis prevalence data from a number of sources including the Global AIDS Monitoring system (GAM) database^14^, databases compiled by WHO and by IHME, data identified from other literature reviews^12,14–21^ and data shared during country applications of the Spectrum-STI tool^22–27^ (see details in SI2). The version used in the present analysis includes data identified and compiled as of 2^nd^ May 2018.

The analysis in this paper is based on the subset of the studies in the database that met the following criteria: (i) the population could be considered representative of the general population (e.g. pregnant women, women at delivery, women attending family planning clinics, and individuals selected for participation in a Demographic and Health Survey); (ii) specimens were collected between 1990 and 2016, and (iii) syphilis was diagnosed using either a non-treponemal or treponemal serological test, or both. Studies conducted among the following non-representative groups were excluded: patients seeking care for an STI or genital symptoms, women with abnormal Papanicolaou smears, women attending gynaecology or sexual health clinics, remote or indigenous populations, men who have sex with men (MSM), and female sex workers (FSW).

All duplicate data points were removed. Studies with a reported prevalence of 0% were approximated as 1 case divided by 100 times the sample size in order to facilitate the estimation of uncertainty intervals. For studies that provided data for two or more years the data were entered separately by calendar year and each year was counted as one data point. When separation by calendar year was not possible, the study was entered as one data point at the mid-point of the study period.

In addition, to generate estimates for overall national women’s populations, we increased the weighted prevalence estimates by 10% to reflect under-sampling of higher-risk populations in ANC and general population surveys, as was done by WHO in their regional and global estimates^4^.

### Adjusting for diagnostic test

All eligible data were adjusted to account for the diagnostic test used in the study. We defined probable active syphilis as concurrent positivity on a non treponemal and treponemal test. This is the definition recommended and used by the WHO^4^ and by the IHME^5^. Prevalence data from studies that used either a treponemal or non-treponemal test alone were adjusted using a method described in previous meta-analyses^12,28–30^: prevalence values for studies using only treponemal or non-treponemal tests without confirmatory tests were multiplied by 0.53 and values from studies where the diagnostic test was unknown by 0.75^15,26–28^. Values based on Rapid treponemal-based test were multiplied by 0.70, as these tests are believed to be relatively specific compared to conventional treponemal tests^6^. Each adjustment multiplier was assumed to have a standard error of ±25%.

### Country eligibility

Prevalence trends were generated for the subset of countries in the Spectrum syphilis database that met the study inclusion criteria and had at least one data point post-2010 and three or more data points from 2000 or later.

### Generating national estimates

All of the data from a country were pooled after adjusting for diagnostic tests. When pooling data, the prevalence in pregnant women attending ANC and in adult women in the general population^4^ was assumed to be the same, and the male-to-female ratio was set at 1:1, in keeping with WHO’s 2012 global estimates^4^ and supported by a recent global meta-analysis^29^ of national household and other general population surveys^31–33^. In addition, it was assumed that all qualifying data were representative, i.e. no adjustments were made for possible over-sampling of urban or rural sites, or for urban/rural prevalence differences.

Each data point in a country was assigned a weight to reflect how representative it was of the national population (see Table SI3). The weighted prevalence data were *scaled within each country by* dividing by sample size, so that a study’s sample size did not influence the estimated national prevalence level or trend. Sample size, however, *did* influence the uncertainty ranges obtained using bootstrap resampling, as described above.

### Time trend analysis

The time trend analysis focused on changes in the median prevalence of active syphilis between 2012 and 2016 based on 400 bootstraps per country and on the average annual proportional decline between 2012 and 2016. Countries were grouped into four categories according to the change in their median estimated prevalence from 2012 to 2016 (proportional as well as absolute, so as to cover the public health significance of the change) combined with the precision of the median estimates in those two years:

- **Substantive decrease**: the prevalence decreased, the absolute difference between the median estimated prevalence at 2012 and 2016 was ≥10% proportionally, ≥0.05% as a percentage point difference, and the lower-bound of the 2012 estimate was above the upper-bound of the 2016 estimate;
- **Non-substantive decrease**: the median estimated prevalence decreased from 2012 to 2016, but the country did not meet the criteria above to be classified as substantive decrease;
- **Substantive increase:** the prevalence increased, the absolute difference between the median estimated prevalence at 2012 and 2016 was ≥10% proportionally, ≥0.05% as a percentage point, and the upper-bound of the 2012 estimate was below the lower-bound of the 2016 estimate;
- **Non-substantive increase**: the median estimated prevalence increased from 2012 to 2016, but the country did not meet the criteria above to be classified as substantive increase.

We also assessed the average annual proportional decline over 2012 to 2016, relative to the annual decline rate that would be expected to meet the WHO global strategy target of 90% reduction from 2018 to 2030^1^.

### Sensitivity analysis

Univariate sensitivity analyses were performed to evaluate the influence of key parameters, choices and uncertainties, on the estimated median prevalence across the modelled countries in 2012 and 2016, and on the number of countries in each of the time trend categories. Parameters and choices explored included tightening the eligibility criteria for the trend analysis to those studies conducted only in ANC women (Scenario A), or requiring studies to have one or more data points from 2012 or later rather than 2011 or later (Scenario B). In addition, we explored the effect of reducing the number of years of prevalence data included in the analysis to data from 2005 and later (Scenario C) and to restricting the analysis to only those studies with results from both a treponemal and non-treponemal test (Scenario D). We also explored a variant statistical model where trends were extrapolated to one rather than two years after and before the latest and earliest national data point (Scenario E).

Lastly, we looked at the impact of expanding the study entry criteria to include countries with prevalence data from routine blood donor screening^34–46^ (Scenario F). In this scenario, we assessed time trends in countries that did not meet the default country eligibility criteria, but that could be estimated if the eligibility criteria were expanded to include data from the screening of blood donations from 2011 or later.

## Results

### Syphilis prevalence data

The Spectrum-STI syphilis database, as of 02 May 2018, contained one or more data points for 186 countries (range per country: 1 to 61 data points; median 6 data points; SI5) that met the study entry criteria. Most data were from routine ANC screening (645 of 1,576 data points; 161 million tests out of 268 million total tests; Table 1), followed by ANC surveys (605 data points, 8.4 million tests). Most studies used dual Rapid Plasma Reagin (RPR) and *Treponema pallidum* hemagglutination assay (TPHA) testing (819 data points, 109 million tests), followed by tests of unknown type and RPR testing alone.

**Table 1:** Adult syphilis prevalence data (1990–2016), available in the Spectrum-STI global database.

	Average year	Number of studies, surveys, and years of routine ANC screening	Number of positive samples	Number of samples tested	Unweighted average prevalence (%)
Data type					
ANC routine	2012	645	1,530,591	161,090,586	0.95
ANC survey	2005	605	113,813	8,422,062	1.35
Women survey	2003	93	13,006	2,333,441	0.56
Men survey	2003	78	5,184	247,945	2.09
Men + Women survey	2005	11	64,659	3,039,211	2.13
Blood donors, Women	2007	2	3	693	0.43
Blood donors, Men	2010	12	1,628	250,398	0.65
Blood donors, Men + Women	2012	130	147,715	92,247,011	0.16
Diagnostic test					
RPR + TPHA	2008	819	652,567	108,529,201	0.60
TPHA	2006	71	107,382	12,299,956	0.87
RPR	2007	353	592,635	42,606,321	1.39
Rapid *treponemal*-based assay	2013	79	143,180	10,107,457	1.42
Test unknown	2011	254	380,835	94,088,412	0.40
WHO world region					
African Region	2006	633	1,231,508	41,757,009	2.95
Region of the Americas	2011	276	186,176	25,142,548	0.74
Eastern Mediterranean Region	2010	130	93,804	16,899,247	0.56
European Region	2011	193	72,373	65,822,040	0.11
South-East Asia Region	2008	133	106,639	27,208,629	0.39
Western Pacific Region	2009	211	186,099	90,801,873	0.20
All data points	**2,008**	**1,576**	**1,876,599**	**267,631,346**	**0.70**

Notes to Table 1: Data available as of 02 May 2018.

For WHO world regions, see: (http://www.who.int/about/regions/en/). ANC = Antenatal Care. RPR = Rapid plasma reagin. TPHA = *Treponema pallidum* hemagglutination assay.

**Figure 1:**
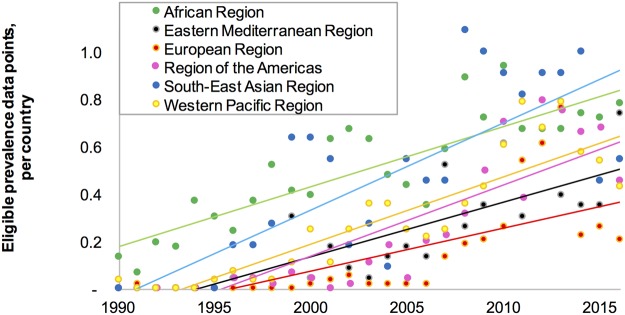
Average number of eligible syphilis prevalence data points, recorded in the Spectrum STI syphilis database, grouped by WHO region. Note to Fig. 1: Lines represent linear trend lines fitted through the data points for each region.

In all world regions, the number of eligible data points per country had generally increased over time (Fig. 1). Most data points were from the WHO African region, while most tests were conducted in WHO Western Pacific region (with fewer data points, of a larger sample size on average).

The data included 60 studies with a reported prevalence of 0%, covering 1.88 million samples tested, i.e. 3.8% and 0.25% of total data points and samples tested.

### National estimates

132 countries met the criteria for time trend analysis. These 132 countries accounted for 78% of the world’s population in 2016.

**Figure 2:**
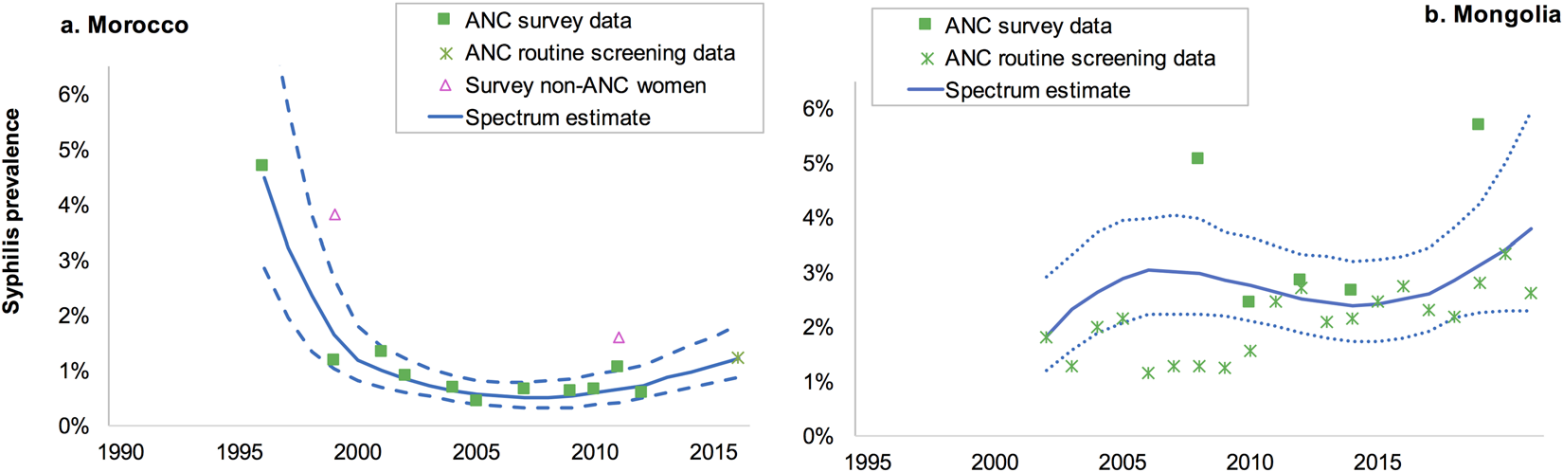
Spectrum-STI estimations of adult female syphilis prevalence (**a**) Morocco and (**b**) Mongolia. Notes to Fig. 2: Data shown are after adjustment for diagnostic test performance. Neither country had any data included from years 1990–1995; both estimations used data from ANC and general populations only; both countries were classified as having a non-substantive prevalence increase over 2012–2016. Solid line = is the best estimate (median of 400 bootstraps), dashed lines are the 95% confidence interval.

Figure 2 shows the Spectrum-STI trend estimates for two of the countries where national Spectrum-STI workshops have been held^22–25^. In Morocco (Fig. 2a) prevalence fell between 2000 and 2007, when Morocco rolled-out syndromic STI management, and has been increasing slowly since 2007. In Mongolia (Fig. 2b), the prevalence increased to 2002, fell slightly between 2002 and 2010 and has been increasing since 2012.

Among the 132 countries, the median national prevalence estimated for 2016 ranged from 0% to 10.0% (as median of 400 bootstraps within each country), with an interquartile range of 0.16% to 1.62% and a cross-country median (of the 132 country medians) of 0.88%. In 2012 the median national prevalence ranged from 0% to 10.0%, the interquartile range was 0.16% to 1.62% and the cross country median was 0.57%.

**Figure 3:**
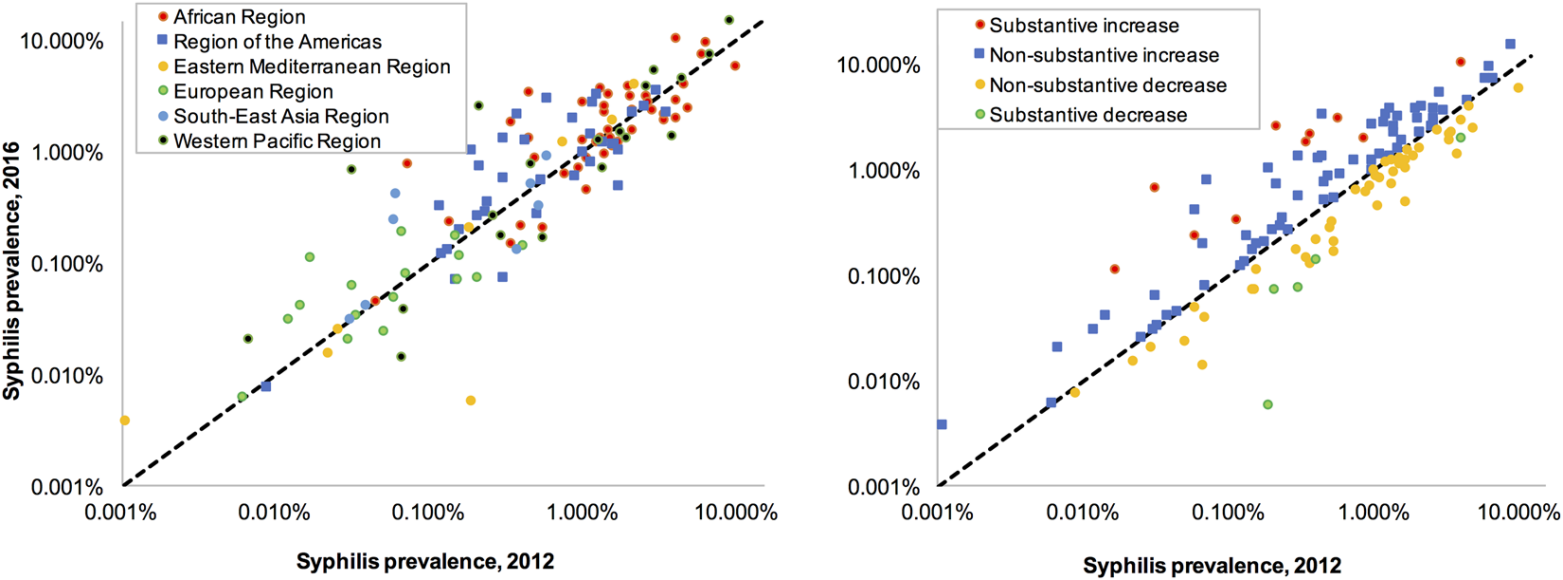
Spectrum-estimated national syphilis prevalence in 2016 as a function of estimated prevalence in 2012, and each country’s time trend classification, for 132 countries: (**a**) Grouped by WHO region. (**b**) Grouped by time trend category. Notes to Fig. 3: The black dotted line indicates equality of prevalence within a country at 2012 and 2016.

Figure 3a shows the median national prevalence of active syphilis in 2012 and 2016 for each country by geographical region. In general, the median prevalence was lowest for countries in the European Region and highest in the African Region. However, there was also considerable variability within regions, especially in the Western Pacific Region. Figure 3b shows the same results, grouped by the time trend category into which each country fell. The prevalence of probable active syphilis between 2012 and 2016 decreased in 54 of the 132 (41%) countries and in 5 of these the decrease was substantive. In the 78 countries where prevalence increased, it was substantive in 10 (Fig. 3b).

Of the 54 countries with a decrease, 12 countries (including 4 of the 5 countries where the decrease was substantive) had a proportional decrease between 2012 and 2016 greater than 54%, the rate corresponding to the average decrease required to achieve the WHO-target of a 90% reduction in the incidence of syphilis globally between 2018 to 2030^1^.

**Figure 4:**
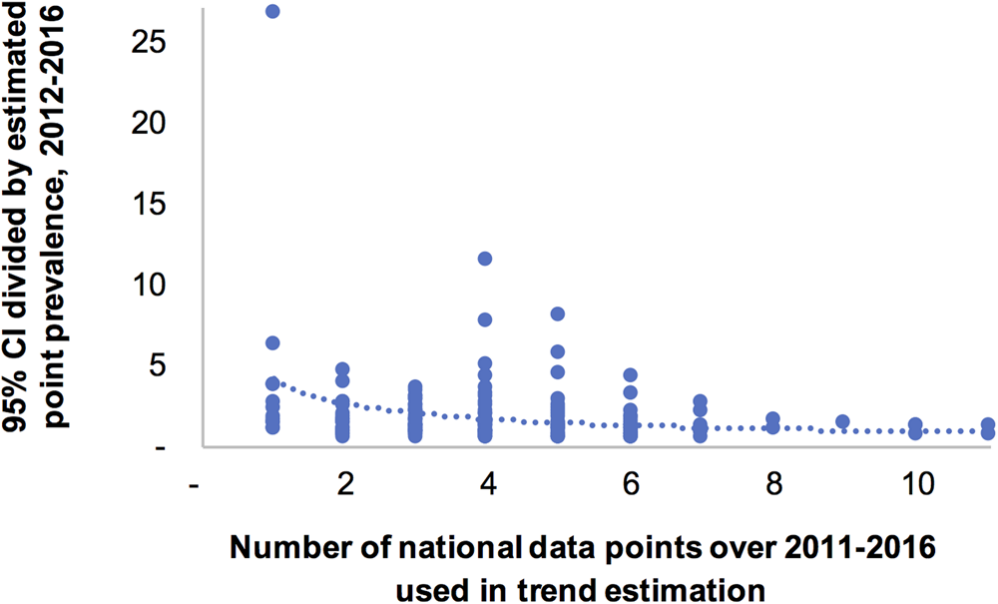
Relation between number of national prevalence data points, and precision of point prevalence estimates. Notes to Fig. 4: Precision expressed, on the y-axis, as the width of the 95% CI (averaged between 2012 & 2016) divided by the point estimate. Each dot represents a country with a Spectrum national trend estimate based on data from ANC and general populations.

The precision of the prevalence estimates improved as the number of country data points between 2011 and 2016 increased (Fig. 4; Pearson R^2^ = 0.05, p = 0.011). Countries with fewer than three data points between 2011 and 2016 had considerably wider 95% CIs, which reduced the statistical significance of any difference in the 2012 to 2016 period.

### Sensitivity analysis

Altering the eligibility of countries in the trend analysis by restricting the analysis to countries with one or more data point post-2011 (Scenario A) had almost no effect on the cross-country median prevalences or on the proportion of countries where prevalence decreased (Table 2). Similarly, changing the statistical model to constrain the extrapolation past the last data point to only one year instead of two years (Scenario B) had no discernible impact on the key outcomes.

**Table 2:** Sensitivity analysis: Spectrum-estimated syphilis prevalence in 132 countries, under varying scenarios of data included and modelling assumptions.

Scenario	Countries included	Median prevalence, 2012	Median prevalence, 2016	Countries with prevalence trend from 2012 to 2016			
				Substantive increase	Non-substantive increase	Non-substantive decrease	Substantive decrease
**Default estimates**	**132**	**0**.**57%**	**0**.**88%**	**14 (11%)**	**64 (48%)**	**48 (36%)**	**6 (5%)**
A. At least one data point from 2012 or later	129	0.55%	0.87%	14 (11%)	62 (48%)	47 (36%)	6 (5%)
B. More restrained time trends: extrapolate past the last year with national data, for 1 year instead of 2 years	132	0.57%	0.92%	13 (10%)	65 (49%)	48 (36%)	6 (5%)
C. Restrict data to 2005 and later (instead of 1990)	130	0.46%	0.93%	17 (13%)	61 (47%)	44 (34%)	8 (6%)
D. ANC (survey & routine screening) data only	131	0.56%	0.90%	18 (14%)	62 (47%)	43 (33%)	8 (6%)
E. Syphilis infections positive on both treponemal and non-treponemal tests	85	0.94%	0.86%	8 (9%)	32 (38%)	42 (49%)	3 (4%)
F. Countries not included in default but made eligible for trend analysis by adding blood donor data	34*	0.04%	0.05%	3 (9%)	21 (62%)	9 (26%)	1 (3%)

Notes to Table 2. The presented medians, which are unweighted, should not be interpreted as indicative of global burden trends, as global trends depend on national population sizes. Scenario F presents 34 countries that were not included in the default analysis.

In none of the 7 scenarios, none of the countries and none of the years between 2012–2016 was the 20% maximum value imposed on estimated prevalence ever reached, neither in the best estimate (i.e. the median of 400 bootstraps) nor in the upper-bound limit of each corresponding 95% confidence interval.

Reducing the number of years of prevalence data included in the analysis (Scenario C) lowered the cross-country median prevalence in 2012 from 0.57% to 0.46% and slightly increased it in 2016 (0.88% to 0.93%). The total number of countries with increasing and decreasing trend was similar, although the proportion of countries where this increase or decrease was substantive became larger. Similarly, constraining the data used to ANC populations, increased the proportion of countries where the trend of increase or decrease was substantive (Scenario D).

Trend results were sensitive to restricting the entry criteria to only surveys where blood samples were dual test positive (i.e. positive on both treponemal and non-treponemal tests; Scenario E). The total number of countries in this analysis was 85 and the cross-country median prevalence in 2012 increased from 0.57% (Default Scenario) to 0.94%, whilst the median prevalence in 2016 increased slightly from 0.88% to 0.86%. This subset of higher-prevalence countries had a larger proportion with a decreasing trend (53%, compared to 41% in the default).

Blood donors were not included in the analysis because of their likely non-representativeness of overall populations; transfusion services often exclude donors with self-reported risks or observed infections. We did, however, examine the subset of countries where there were insufficient data for estimation according to the default eligibility criteria, but where a time trend could be estimated if we included prevalence data from routine blood donor screening^34–46^ (Scenario F). Thirty-four countries met this criteria and their cross country median prevalence was much lower (0.04–0.05% from 2012 to 2016), and the proportion of countries with a decreasing prevalence was also lower (29%) than in the default (41%). These were mainly high-income and higher-middle-income countries, mostly in the European Region, and secondarily in the Western Pacific and Eastern Mediterranean Regions; they were also mostly smaller countries with smaller adult populations somewhat smaller countries with adult populations of on average also mainly smaller-populations (SI4 Table).

## Discussion

The Spectrum-STI estimation model is a useful tool, developed for national health officials to explore historic trends in adult syphilis prevalence to inform and improve their program planning, target-setting and progress evaluation in syphilis control and congenital syphilis elimination. The present study is the first attempt to examine systematically *recent trends* in the prevalence of syphilis at the national level, and collectively for much of the world’s population. The analysis complements previous efforts that looked at trends over time in specific countries^30^ or at regional and global levels^47,48^.

The Spectrum estimates indicate that many countries are making progress in reducing the prevalence of syphilis in adult women. The estimated prevalence in 2016 was lower than in 2012 in 54 of the 132 countries with sufficient data to meet our study entry criteria, and in 5 countries this decrease was substantive from a public health perspective. In the 78 countries where the prevalence was higher in 2016 than in 2012 the increase was substantive in 10. At the current rate of declines only 12 of the 132 countries are on course, assuming this decline continues at the same rate, to meet the WHO target of a 90% reduction in the incidence of syphilis in adults from 2018 to 2030^1^.

Seventy-four countries had insufficient data to meet the criteria for generating a Spectrum estimate. For 34 of these, by expanding the study entry criteria to include blood donors, we were able to generate a trend estimate; this subset of countries had a much lower prevalence of infection. Of the remaining countries, 20 had at least one data point and these had observed prevalences lower than countries in the default and blood donor-based estimates (SI4). For another 20 countries we were not able to identify any data post-1999 from a study in an ANC or general population; these were primarily high-income countries in the European and Eastern Mediterranean regions where ANC women are screened for syphilis but the data are either not collated for surveillance purposes, or not reported through international mechanisms like the Global AIDS Monitoring system (see SI4 and SI5).

Restricting the analysis to those studies where individuals were tested with both treponemal and non-treponemal tests (n = 85) resulted in an increasing proportion of countries with declining rates from 41% to 53%. This different result is in part due to a selective, higher-prevalence set of countries (with the countries with higher prevalence at 2012 more often showing a subsequent decline, Fig. 3b), and it may in part reflect a more rigid data set less affected by uncertainties and potential mis-classifications in diagnostics tests and their adjustment factors (which were based on a meta-analysis of studies pre-2014^28^). The larger declines estimated for the higher-prevalence countries also illustrate that at regional and global levels syphilis may still be declining, even if equal number of countries (but more often, less populous and lower-prevalence countries) showed increasing trends.

At a country level the Spectrum-STI syphilis estimates concur with recent historic trend estimates, for those few countries with such an independent estimate available^30^. At regional level, a recent meta-regression of the Spectrum database (in a year’s older, less complete version), with data aggregated across countries, estimated average proportional annual decline rates over 1990 to 2016 of around 5% for the African Region, 8% for the Region of the Americas, 16% for the Eastern Mediterranean Region, 6% for the European Region, 10% for the South-East Asian Region and 3% for the Western Pacific Region^29^, which are consistent with our Spectrum-based estimates for countries in these regions. These prior syphilis trend analyses^29,30^ have generally highlighted a declining trend over longer time horizons, reflecting behavioral risk reductions (e.g. increased use of condoms and fewer partners)^49,50^, improvements in the coverage of ANC-based syphilis screening and treatment as well as treatment of partners^48^, and increased use of antibiotics such as oral penicillin, tetracyclines and macrolides, commonly prescribed for skin, respiratory, and other non STI-infections, which have activity against syphilis as well^51^. This includes over 741 million doses of azithromycin administered as part of mass trachoma treatment campaigns since 1999 in trachoma-endemic countries^52^.

Our analysis shows that in many other, mainly lower-prevalence countries, syphilis prevalence tended to increase from 2012 to 2016. Possible reasons for recent increases in these countries may include: saturation of prior declines associated with roll-out of ANC-based services; fatigue with safer sex and behaviour-based HIV prevention, possibly in response to the rapid expansion of access to HIV treatment drugs; shifts from condom usage to longer-term hormonal contraception^53^; and the recent global shortage of benzathine-penicillin used as first-line treatment for syphilis^54^. Furthermore, measuring prevalence using RPR/TPHA dual testing or RPR-based testing may obscure recent declines owing to the time lag for a person to test RPR negative after treatment. For example, if the median time for RPR to sero-revert after treatment is 1 to 2 years^55–57^ real prevalence reductions will only become apparent after a 1 to 2 year delay.

### Limitations

The quality of statistical trend estimates reflects the quantity, quality and representativeness of the input data. The Spectrum syphilis database draws on data from a variety of sources including the Global AIDS Monitoring System and is the most global comprehensive database on syphilis now. Experience from country workshops^22,25–27^, however, suggests that there are additional data available within countries. Country outreach can also improve data quality, for example, by providing information on the specific diagnostic test types.

The Spectrum estimates were based on data primarily from ANC women (79% of data points, 63% of tests). The focus on data from ANC women means we may be over-estimating the prevalence in the general female population, as ANC women are sexually active and younger. Also recent syphilis declines may be larger in ANC populations than the overall adult population, as multiparous pregnant woman may have been diagnosed and treated in an earlier pregnancy. The HIV field has noted that ANC sentinel surveys and ANC-based HIV screening historically over-estimate HIV prevalence compared to general population surveys, as sentinel sites and routine screening were usually first established in urban, higher-risk areas^58,59^; however, syphilis prevalence has not consistently been higher in urban areas, and may in fact be higher in underserved rural populations, as a function of access to health care and exposure to antibiotics.

Our analysis included a small number of data points from community-based studies in men, and assumed a male-to-female ratio of 1 to 1, based on a recent global meta-analysis^29^. Removing these data points had almost no impact on the results (sensitivity analysis, Scenario D). The lack of male data, however, is a considerable challenge when generating national syphilis burden estimates. The few countries with data highlight that there is considerable variation in the male-to-female ratio between countries and within countries.

The Spectrum-STI model is a work in progress and, like the Spectrum HIV models, is being refined as more data become available. The current version of the model is focused on modeling trends over time in the general population—data from populations at higher risk of infection are not incorporated. A new variant of Spectrum-STI is under development that will include sub-group estimates such as for FSW and MSM, incorporating data from other sources like integrated bio-behavioural surveillance surveys (see^26^ for an example of sub-groups estimation). This will make it possible to remove the 10% adjustment factor for high-risk populations from the current version of Spectrum-STI. For Morocco, Mongolia and Colombia, comparative prevalences of syphilis in FSW relative to Spectrum-STI estimates for women, combined with national estimates of numbers of FSWs^22,25,27^, suggested that a 10% uplift in prevalence was a reasonable adjustment for them. However, contributions of higher-risk populations vary across countries and may be higher, in countries with low STI prevalences^26^. Several higher-income countries^60,61^ as well as China^62^ recently recorded rising syphilis prevalence in MSM, which may be driving reversals from past prevalence declines among men and in the general adult population^63^. Other refinements that could be considered for future Spectrum versions include sub-national disaggregation and looking in more detail at age. For large countries like India, Indonesia, China, Nigeria and the Democratic Republic of the Congo, national aggregation is a limitation^62,64^.

## Conclusion

Spectrum-STI provides a useful tool for countries to monitor trends over time in the prevalence of syphilis. The quality and usefulness of these estimates reflects both the quality and quantity of the data available. As countries improve their syphilis surveillance, estimations should also improve and countries then should be in a better position to set targets for syphilis control and elimination, and to mobilize funding to expand access to existing services and launch and intensify complementary services, such as screening and treatment for key populations. The trend analysis presented in this paper suggests that whilst the prevalence of syphilis fell in a number of countries between 2012 and 2016, the prevalence increased in more countries than it fell. This highlights the need to intensify efforts to improve syphilis surveillance and control if countries are to meet the WHO’s syphilis reduction targets for 2030.

## Electronic supplementary material

Supplementary Information files 2, 3 and 4

Supplementary Dataset 5

Supplementary Dataset 1
