# Supplementary material for: Syphilis prevalence trends in adult women in 132 countries – estimations using the Spectrum Sexually Transmitted Infections model
Source: Sci Rep. 2018 Jul 31;8:11503. doi: 10.1038/s41598-018-29805-9 (PMC6068092; doi:10.1038/s41598-018-29805-9)
Supplement: Supplementary file 1 — Supplementary Information files 2, 3 and 4 [file 41598_2018_29805_MOESM1_ESM.docx]

**Supplementary Information files, accompanying manuscript:**

**Syphilis prevalence trends in adult women in 132 countries– estimations using the Spectrum Sexually Transmitted Infections model**

Eline L. Korenromp^1*^, S. Guy Mahiané^2^, Nico Nagelkerke^3^, Melanie Taylor^4,5^, Rebecca Williams^6^, R. Matthew Chico^6^, Carel Pretorius^2^, Laith J. Abu-Raddad^7^ & Jane Rowley^8^

*Version 11 June 2018*

**SI2. The Spectrum STI Syphilis Database**

The Spectrum STI syphilis database includes data from:

1. Global AIDS Monitoring system, collated in the WHO’s global health observatory[^1^](#_ENREF_1), on prevalence of syphilis among women screened at first ANC visit (or if first visit not available, any visit), as reported by national health ministries, from either routine ANC screening results, or nationally representative sample surveys (of first ANC visits if available; otherwise any visit) up to 2016;
2. WHO’s database of (sub-)nationally representative general population STI prevalence surveys in low-risk men and/or women, compiled for the 2005 and 2008 global estimates[^2^](#_ENREF_2)^,^[^3^](#_ENREF_3);
3. The IHME, University of Seattle, database compiled for the 2015 Global Burden of Disease study (accessed, 29 November 2017), based on a PubMed search and testing data from reports in the Global Health Data Exchange (GHDx) database[^4^](#_ENREF_4). This included data from ANC and general adult populations;
4. Other representative measurements identified through systematic reviews of STI prevalences in ANC women in sub-Saharan Africa[^5^](#_ENREF_5), North Africa and the Eastern Mediterranean (as part of the regional Middle East and North Africa HIV Epidemiology Synthesis Project database)[^6^](#_ENREF_6)^,^[^7^](#_ENREF_7) and globally[^8^](#_ENREF_8)^,^[^9^](#_ENREF_9), and from published national surveillance reports compiled by Ministries of Health of countries which previously conducted national Spectrum-STI estimations[^10-13^](#_ENREF_10);
5. National HIV/syphilis household surveys conducted in 2016 in Zimbabwe[^14^](#_ENREF_14), Uganda[^15^](#_ENREF_15) and Zambia[^16^](#_ENREF_16).

The database includes data for 186 countries as of 02 May 2018. Table SI4 compares the 132 countries included in the default trend analysis, the 54 countries in the data base that were not included or included only as a sensitivity analysis, and the 20 countries that had no data. Over half of the 20 missing countries were from the European Region, where most countries test for syphilis in ANC (estimated 95% plus coverage), but very few countries collate this data routinely at the national level [^17^](#_ENREF_17). The 20 countries with at least one data point but less than 3 had, on average, lower observed prevalences (aggregate 0.045%) compared to the estimated countries (aggregate 0.726%), and their data were from on average earlier years.

**SI3 Table. Calculation of weights for the Spectrum estimation, for prevalence data points based on population sampled**

| **Population** | **Study Design** | **Weight** |
| --- | --- | --- |
| ANC | Nation-wide survey (cluster design) – all sites | 100% |
| ANC | Nation-wide survey (cluster design) – subset of sites | Number of sites sampled, divided by maximum number of sites |
| ANC | Routine screening | Number of ANC women tested, divided by number of ANC women registered by the country* |
| General adult population | Nationally representative survey (e.g. DHS) – 20 or more sites | 100% |
| General adult population | Nationally representative survey – fewer than 20 sites | Number of sites divided by 20 (e.g. 15% for a 3-sites study) |

Notes to SI3 Table.

* The denominator was based on the coverage of routine syphilis screening among women presenting for first ANC visit (or, if first visit not available, any ANC visit), among pregnant women attending for first ANC visit. Sample sizes were imputed for a few ANC routine data points: (1) If data were available for the years both before and after the year with missing sample size, we imputed the missing sample size linearly from sample sizes in the two years with data; (2) If data were not available from before the data point with missing sample size, this sample size was imputed as 50% of the sample size of the first next year with a sample size specified.

**SI4 Table. Comparison of countries that met the study entry criteria (3 or more data points from 2000 to 2016 of which at least one post-2010) and those that did not.**

| **Group of countries, by data availability** | | **Estimated, default scenario** | **Included (only) in sensitivity analysis, scenario F, including blood donor data** | **Insufficient data for time trend analysis** | **No data** |
| --- | --- | --- | --- | --- | --- |
| Countries | | 132 | 34 | 20 | 20 |
| Total population of women 15-49 years, 2016 | | 1,468 million (79%) | 256 million (14%) | 129 million (7%) | 16 million (1%) |
| Data points | | 1,382 | 162 | 37 |  |
| N positive (all data points) | | 1,753,591 | 119,655 | 9,689 |  |
| N tested (all data points) | | 177,751,531 | 81,891,845 | 21,476,372 |  |
| Observed prevalence % (all data points*) | | 0.99% | 0.15% | 0.05% |  |
| Countries, by WHO region | African Region | 44 | 1 | 1 | 0 |
|  | Region of the Americas | 35 | 1 | 1 | 7 |
|  | Eastern Mediterranean | 10 | 4 | 5 | 4 |
|  | European Region | 16 | 18 | 12 | 8 |
|  | South-East Asia Region | 8 | 2 | 1 | 0 |
|  | Western Pacific Region | 19 | 8 | 0 | 1 |
| Countries, by income group | High income | 9 | 17 | 9 | 7 |
|  | Upper middle income | 46 | 8 | 5 | 5 |
|  | Lower middle income | 46 | 7 | 4 | 3 |
|  | Low income | 31 | 0 | 2 | 0 |
|  | No income category assigned | 0 | 2 | 0 | 5 |
| First year of data: median (and range) across countries | | 2006 (1990-2013) | 2011 (1990-2012) | 2011 (1998-2013) |  |
| Last year of data: median (and range) across countries | | 2016 (2011-2017) | 2013 (2013-2017) | 2012 (2001-2015) |  |

Notes to SI4 Table.

* Unweighted sum across data points.

Country income classification from the World Bank list of economies, June 2017 (databank.worldbank.org/data/download/site-content/CLASS.xls ).

**References for the Supplementary Information files:**

1 World Health Organization. Global health observatory indicator data: Antenatal care (ANC) attendees tested for syphilis at first ANC visit. (World Health Organization,, Geneva, 2017). <<http://apps.who.int/gho/data/node.imr.PerctestedANC?lang=en>>

2 World Health Organization. Prevalence and incidence of selected sexually transmitted infections -- Chlamydia trachomatis, Neisseria gonorrheae, syphilis and Trichomonas vaginalis. Methods and results used by WHO to generate 2005 estimates. (Geneva, 2011). <<http://apps.who.int/iris/bitstream/10665/44735/1/9789241502450_eng.pdf>>

3 World Health Organization. Global incidence and prevalence of selected curable sexually transmitted infections -- 2008. (Geneva, 2012). <<http://apps.who.int/iris/bitstream/10665/75181/1/9789241503839_eng.pdf>>

4 Institute for Health Metrics and Evaluation. Global Health Data Exchange (GHDx) database. (Seattle, 2017). <<http://ghdx.healthdata.org/>>

5 Chico, R. M. *et al.* Prevalence of malaria and sexually transmitted and reproductive tract infections in pregnancy in sub-Saharan Africa: a systematic review. *JAMA* **307**, 2079-2086, doi:10.1001/jama.2012.3428 (2012).

6 Abu-Raddad, L. J. *et al.* Epidemiology of HIV infection in the Middle East and North Africa. *AIDS* **24 Suppl 2**, S5-23, doi:10.1097/01.aids.0000386729.56683.33 (2010).

7 Abu-Raddad, L. J. *et al.* *Characterizing the HIV/AIDS epidemic in the Middle East and North Africa: Time for Strategic Action*. (The World Bank Press, 2010).

8 Joseph Davey, D. L. *et al.* Prevalence of Curable Sexually Transmitted Infections in Pregnant Women in Low- and Middle-Income Countries From 2010 to 2015: A Systematic Review. *Sex Transm Dis* **43**, 450-458, doi:10.1097/OLQ.0000000000000460 (2016).

9 Williams, R. *et al.* The Sustainable Development Goals and curable sexually transmitted infections among pregnant women: A systematic review and meta-analysis. (manuscript in preparation).

10 Korenromp, E. L. *et al.* Estimating prevalence trends in adult gonorrhoea and syphilis prevalence in low- and middle-income countries with the Spectrum-STI model: results for Zimbabwe and Morocco from 1995 to 2016. *Sex Transm Infect* **sextrans-2016-052953**, doi:10.1136/sextrans-2016-052953 (2017).

11 Enkhbat, E. *et al.* (ed Mongolian Field Epidemiology Training Department of NCCD).

12 Korenromp, E. L. *et al.* Adult syphilis, chlamydia and gonorrhea prevalence and incidence, and congenital syphilis incidence in Colombia, 1995-2016 – estimates using the Spectrum-STI model. *Pan-American journal of Public Health* **in press** (2018).

13 Rowley, J. & Korenromp, E. L. A pilot application of the Spectrum-STI model in a low-prevalence setting: Estimation of STI prevalence and incidence trends in Georgia. Technical Report, based on a workshop in Tbilisi, Georgia, August 23-24th 2017. (London, 2017). <https://spectrummodel.zendesk.com/hc/en-us/articles/115003492452-Georgia-Spectrum-STI-estimation-2017-final-report & https://spectrummodel.zendesk.com/hc/en-us/articles/115003469011-Georgia-Spectrum-STI-estimation-2017-annex-to-final-report >

14 ICAP project at Columbia University, Centers for Disease Control USA, Zimbabwe National AIDS Council (NAC), Zimbabwe National Statistics Agency (ZIMSTAT) & Zimbabwe Biomedical Research and Training Institute (BRTI). Zimbabwe population-based HIV impact assessment ZIMPHIA 2015-2016. Fact sheet. (Washington DC, 2016). <<http://phia.icap.columbia.edu/wp-content/uploads/2016/11/ZIMBABWE-Factsheet.FIN_.pdf>>

15 ICAP project at Columbia University, Centers for Disease Control USA & Westat. Uganda population-based HIV impact assessment UPHIA 2016-2017. Summary sheet: preliminary findings. (Washington DC, 2017). <<http://phia.icap.columbia.edu/wp-content/uploads/2017/09/UPHIA-Uganda-factsheet_A4.new_HR.pdf>>

16 Zambia Ministry of Communicable Diseases Maternal and Child Health *et al.* Zambia Population-based HIV impact assessment (ZAMPHIA) 2016. First report. (Lusaka, 2017). <<http://phia.icap.columbia.edu/wp-content/uploads/2017/11/FINAL-ZAMPHIA-First-Report_11.30.17_CK.pdf>>

17 European Center for Disease Prevention and Control. Antenatal screening for HIV, hepatitis B, syphilis and rubella susceptibility in the EU/EEA. A Member State survey of policies and practices in the prevention of mother-to-child transmission Report No. TQ-01-16-294-EN-N, (Stockholm, 2016). <https://ecdc.europa.eu/sites/portal/files/media/en/publications/Publications/antenatal-screening-HIV-hepatitis-B-syphilis-rubella-EU.pdf>
