# Supplementary material for: Syphilis prevalence trends in adult women in 132 countries – estimations using the Spectrum Sexually Transmitted Infections model
Source: Sci Rep. 2018 Jul 31;8:11503. doi: 10.1038/s41598-018-29805-9 (PMC6068092; doi:10.1038/s41598-018-29805-9)
Supplement: Supplementary file 3 — Supplementary Dataset 1 [file 41598_2018_29805_MOESM3_ESM.zip › Syph_Analysis_SampleCode_F/SoSSyphTrends_0.1.0.zip!/SoSSyphTrends/html/00Index.html]

R: Estimates Syphilis countries's prevalence and incidence trends

# Estimates Syphilis countries's prevalence and incidence trends

---

## Documentation for package ‘SoSSyphTrends’ version 0.1.0

- DESCRIPTION file.

## Help Pages

|  |  |
| --- | --- |
| BestFit2PIDLLik\_Random | Fit bootstrapped prevalence data |
| BestFitPIDLLik\_Random | Fit prevalence data |
| fCountryAnalysis\_glob | Prevalence and incidence estimation |
| fCountryAnalysis\_globN | Prevalence and incidence estimation |
| FitSegPol2PIDLLik\_Random | Optimization of prevalence model parameters |
| GFitSegPol2PIDLLik\_Random | Optimization of prevalence model parameters |
| GMOptimSegPolDF | Minimization of function based on Nelder-Mead. |
| InciSegPol2PID | Incidence calculation |
| LLikSegPol2PID\_Random | Negative log-likelihood calculation |
| logit | Inverse of the expit function |
| nLBCheckCalc | Check that the minimum incidence is within the bounds. |
| nUBCheckCalc | Check that the maximum incidence is within the bounds. |
| PrevRandEff | Prevalence calculation |
| ProjSegPol2PID | Prevalence calculation |
| SSSegPol2PID\_Random | Sum of square of prevalence calculation |
